# Sequential transarterial chemoembolization and early radiofrequency ablation improves clinical outcomes for early-intermediate hepatocellular carcinoma in a 10-year single-center comparative study

**DOI:** 10.1186/s12876-021-01765-x

**Published:** 2021-04-20

**Authors:** Liangliang Yan, Yanqiao Ren, Kun Qian, Xuefeng Kan, Hongsen Zhang, Lei Chen, Bin Liang, Chuansheng Zheng

**Affiliations:** 1 Department of Radiology, Union Hospital, Tongji Medical College, Huazhong University of Science and Technology, Wuhan, 430022 China; 2 Hubei Key Laboratory of Molecular Imaging, Wuhan, 430022 China

**Keywords:** Transarterial chemoembolization, Early radiofrequency ablation, Hepatocellular carcinoma, Overall survival, Time to tumour progression

## Abstract

**Background:**

Transarterial chemoembolization (TACE) and radiofrequency ablation (RFA) are effective treatment methods for unresectable hepatocellular carcinoma (HCC). However, there is still a lack of clinical research on whether early sequential RFA, compared with late combination therapy, can improve the long-term efficacy of initial TACE treatment.

**Methods:**

This retrospective study investigated a cohort of patients who underwent combination therapy using TACE and RFA (TACE followed by RFA) from January 2010 to January 2020 at our medical centre. A total of 96 patients underwent TACE combined with early RFA (usually during the first hospitalization), which was called TACE + eRFA. Thirty-four patients received 1–2 palliative TACE treatments first and then underwent TACE treatment combined with late RFA (TACE + lRFA). All patients continued to receive palliative TACE treatments after intrahepatic lesion progression until reaching intolerance. The overall survival (OS) rate, time to tumour progression (TTP), tumour response rate and major complication rates were compared between the two groups.

**Results:**

There were significant differences in the median OS (46 months vs 33 months; *P* = 0.013), median TTP (28 months vs 14 months; *P* < 0.00), objective response rate (ORR) (89.6% vs 61.8%, *P* = 0.000) and disease control rate (DCR) (94.8% vs 73.5% *P* = 0.002) between the two groups. Multivariable analysis revealed that the Barcelona Clinic Liver Cancer stage was an independent risk factor for OS. Meanwhile, multivariable analysis revealed that TACE + eRFA was associated with an enhanced TTP.

**Conclusion:**

Early sequential RFA treatment in patients with early-intermediate HCC can improve local tumour control and clinical outcomes while reducing the frequency of TACE treatment. In clinical practice, in HCC patients initially treated with TACE, it is recommended to combine RFA as soon as possible to obtain long-term survival.

## Introduction

Cancer is the first or second cause of premature death (30–69 years old) in 134 countries worldwide [1]. Liver cancer is a common malignancy of the digestive system, and hepatocellular carcinoma (HCC) is the main pathological type of liver cancer and the fourth most common cause of cancer-related death [2]. HCC exhibits an annual prevalence growth trend [3–5]. The international and authoritative Barcelona Clinic Liver Cancer (BCLC) staging system is applied according to the patient's tumour characteristics, liver function and physical status and is widely accepted [6, 7]. The implementation of surveillance programs for high-risk populations and advances in imaging-based diagnostic technology have increased the rate of early HCC diagnosis [8]. Through novel surgical methods, such as orthotopic liver transplantation or liver resection, early HCC can be cured [9]. Unfortunately, only a portion of HCC patients are suitable for surgical therapies due to an unfavourable tumour location, the presence of multiple tumours, a poor hepatic reserve and a shortage of donor livers. The 5-year recurrence rate in HCC patients treated with liver resection is as high as 50–70% [8–10].

Radiofrequency ablation (RFA) is the first-line ablation technique applied through an electrode tip inserted into the target lesion that induces coagulation necrosis and is a widely accepted treatment option for patients with early-stage HCC [11]. Compared with liver resection or liver transplantation, RFA can provide similar survival odds [9, 12]. However, due to the heat-sink effect that occurs next to large blood vessels, the postoperative recurrence rate is still high, and RFA has a poor curative effect in HCC patients with a high tumour burden [13, 14].

In recent years, transarterial chemoembolization (TACE) has been widely used as a palliative therapy in the treatment of HCC patients who are unsuitable for radical therapies [15]. TACE can cause partial necrosis of tumour cells through a strong cytotoxic effect combined with ischaemia [16]. However, not all feeding arteries can undergo chemoembolization by TACE, since they consist of multiple nodes and can develop de novo collateral arteries [17, 18]. Local recurrence accounts for the majority of relapses following TACE treatment. However, following repeated TACE sessions, TACE failure/refractoriness often occurs, resulting in decreased overall survival (OS) [19]. Consequently, effective adjuvant therapy is essential to prevent or delay relapses.

Recently, studies have indicated that RFA combined with TACE may improve the local control of tumours with a diameter of over 3 cm [20, 21]. However, there have been no reports on the timing of the combination. Consequently, this study analysed whether early sequential RFA treatment, following initial TACE, can improve the long-term efficacy against HCC in such patients compared with late combination treatment.

## Methods

### Study design and patient selection

This was a retrospective study performed in accordance with local and national laws and abiding by the guidelines of the Helsinki Declaration. Approval for this study was obtained from The Ethics Committee of Tongji Medical College, Huazhong University of Science and Technology. The need for informed consent was waived by The Ethics Committee of Tongji Medical College, Huazhong University of Science and Technology.

From January 2010 to January 2020, 436 consecutive patients underwent combination therapy using TACE and RFA (TACE followed by RFA) at our medical centre. Prior to these patients undergoing initial TACE therapy, the treatment strategy was recommended by the multidisciplinary tumour board. Considering the patient’s age, any serious comorbidities (such as portal hypertension), the patient’s compliance, the tumour location (close to the centre, near large vessels or the diaphragm), the lack of donor livers and the limited efficacy of TACE and RFA alone, some patients were not deemed suitable for liver resection or liver transplantation; these patients were recommended for combination therapy (TACE followed by RFA). The time of RFA following TACE was dependent on the disappearance of symptoms postembolization and the recovery of liver function. In this study, the mean interval between TACE and RFA in the TACE combined with early RFA (TACE + eRFA) group was 6.3 days (1–14 days), and the median interval was 5 days. In the TACE combined with late RFA (TACE + lRFA) group, the mean interval between TACE and RFA was 6.1 days (2–13 days), and the median interval was 5.5 days. There was no significant difference in the interval between the two groups (*P* > 0.05). Prior to undergoing the initial TACE treatment, all patients were informed of the benefits and risks of the combination therapy. TACE + eRFA was defined as early sequential RFA treatment (usually during the first hospitalization) following initial TACE. There were patients who were unwilling to take the risk, could not afford the cost of two treatments, or recognized the efficacy of TACE treatment alone but refused to undergo the initial TACE combination therapy. In these patients, following 1–2 palliative TACE treatments, TACE + lRFA was performed on the target lesion.

The diagnosis of HCC depended on the diagnostic criteria of the European Association for the Study of the Liver (EASL) and the American Association for the Study of Liver Disease (AASLD) [8, 10]. A total of 130 patients met the inclusion criteria and were included in this study. The inclusion criteria consisted of the following: (1) unresectable HCC diagnosed by medical imaging or needle biopsy; (2) Child–Pugh class A or B; (3) number of tumours ≤ 3; (4) Eastern Cooperative Oncology Group (ECOG) score of 0; (5) no evidence of invasion of the portal or hepatic venous branches, extrahepatic metastasis, or uncontrolled ascites; and (6) BCLC stage A/B.

**Fig. 1:**
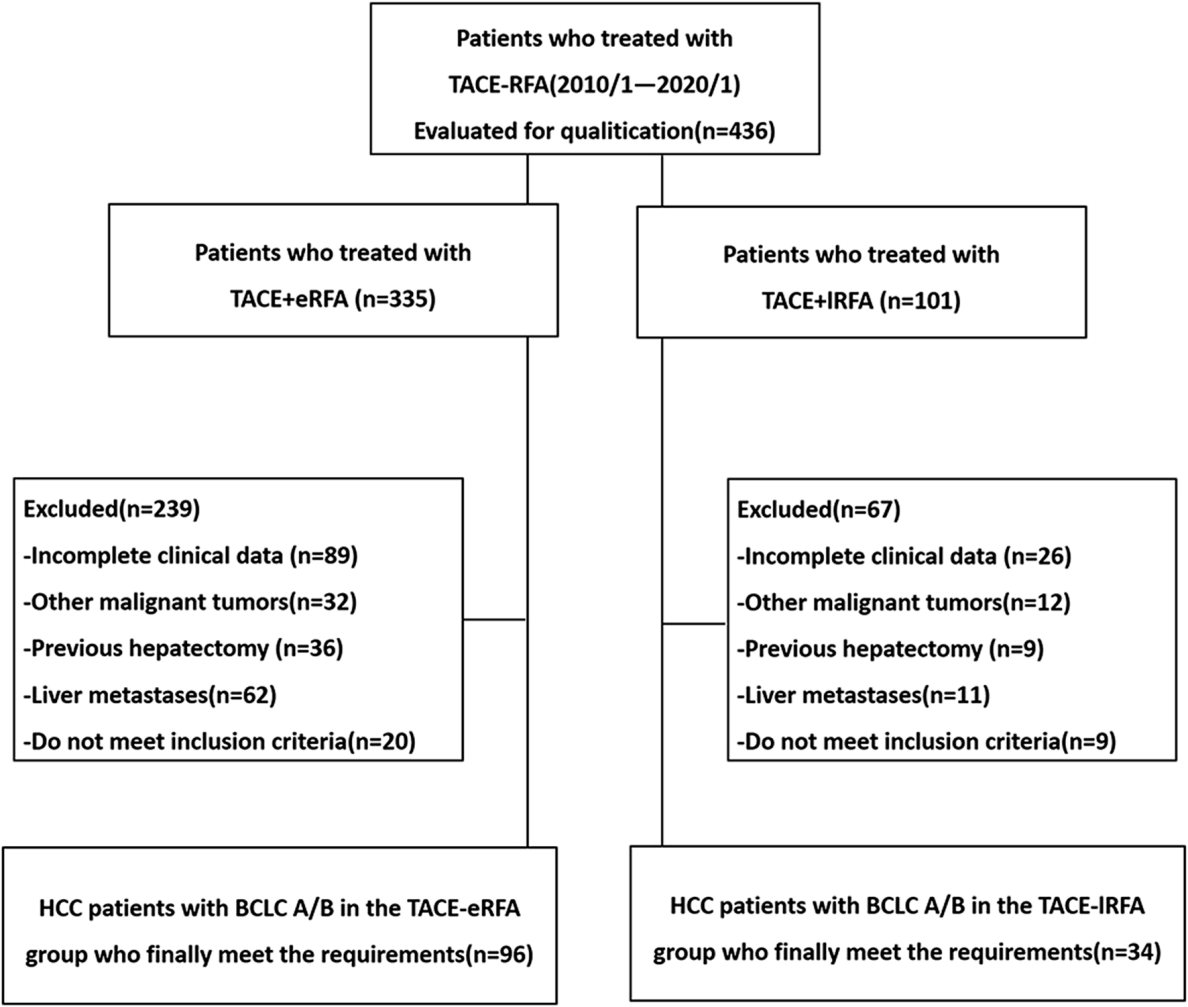
Enrolment of patients. The flow chart shows the screening procedure for patients with Barcelona Clinic Liver Cancer (BCLC) stage A/B hepatocellular carcinoma (HCC)

Patients were excluded if the exclusion criteria were met. The exclusion criteria consisted of the following: (1) any previous treatment for HCC; (2) renal failure, cardiac failure or haemorrhagic risk (< 50 × 10^9^/L); (3) other malignancies or liver metastases; and (4) incomplete clinical data (Fig. 1).

### TACE procedure

TACE was performed according to our institutional standard protocol, which has been previously reported [20]. All operators had at least five years of experience in performing TACE procedures. In essence, tumour staining and tumour-feeding arteries were determined by angiography. Consequently, a 2.6-Fr microcatheter (Terumo™, Japan) was inserted into the tumour-feeding arteries as selectively as possible. First, an emulsion of 2–20 mL of iodized oil (Lipiodol Ultra-Fluid; Laboratoire Andre Guerbet, Aulnay-sous-Bois, France) and 20–60 mg of doxorubicin hydrochloride (Hisun Pharmaceutical Co., Ltd., Zhejiang, China) was administered into the target vessels. The dosage of lipiodol and doxorubicin hydrochloride was determined according to the size/vascularity of the tumour and the patient’s underlying liver function. Typically, according to the blood supply and tumour diameter, the dosage of iodized oil was 1–3 times the tumour diameter (1–3 mL/cm diameter). For patients with a large tumour burden (usually tumours larger than 10 cm) or poor liver function (Child–Pugh score ≥ 8), the initial TACE treatment was performed via fractional embolization or using a reduced amount of emulsion to avoid liver failure. Consequently, gelatine sponge particles (300–500 µm, Cook™, Bloomington, Indiana, USA) mixed with contrast material were administered into the tumour-feeding arteries until arterial flow stasis was achieved.

### Percutaneous RFA technique

The RFA procedure was performed in accordance with the standard treatment regimen described in our previous study [20, 22]. In brief, following analgesia (10 mg of morphine) and local anaesthesia (5–10 mL of lidocaine), the electrode needle was inserted into the tumour nodule under the guidance of ultrasound or computed tomography (CT). The RFA procedure was performed with a 460-kHz RF generator (Rita Medical Systems, Mountain View, California, USA) and a 14-gauge probe/15-cm-long multiple electrode (Rita Medical Systems, Mountain View, California, USA). To attain a safe range of 0.5–1.0 cm, multiple overlapping ablation zones were required.

### Definition and evaluation of data

The time to tumour progression (TTP) and OS rate were compared between the TACE + eRFA and TACE + lRFA groups. The time of combined treatment to disease progression (local progression or intrahepatic/distant metastasis) was defined as the TTP. OS was defined as the interval between the first TACE procedure and either death or the last follow-up. The modified Response Evaluation Criteria in Solid Tumors (mRECIST) was used to evaluate the treatment response at approximately 1–1.5 months after the combined treatment [23]. A complete response (CR) was defined as the absence of enhancement in all target lesions. A partial response (PR) was defined as at least a 30% decrease in the sum of the diameters of viable tumours. Progressive disease (PD) was defined as an increase of at least 20% in the sum of the diameters of the target lesions. Stable disease (SD) was defined as any response that did not qualify as either a PR or PD. Objective tumour regression was defined as a CR or PR. Disease control was defined as a CR, a PR or SD. The Society of Interventional Radiology classification system was also implemented to evaluate the safety of TACE or RFA in both groups [24]. Major complications were defined as events leading to patient death and/or disability.

### Follow-up

Laboratory tests and contrast-enhanced CT or magnetic resonance imaging (MRI) examinations were performed one month after combined treatment. Imaging (contrast-enhanced CT or MRI) and laboratory examinations were performed every 2–3 months for patients. Once local progression or intrahepatic metastasis occurred, palliative TACE treatment was given until it was deemed intolerable by the patient. Follow-up continued until patient death or the end point of this study.

### Statistical analyses

All analyses were performed using SPSS 24.0 software [IBM™, Armonk, New York], and a *P* value of < 0.05 was considered statistically significant. Quantitative data are presented as the mean ± standard deviation, and discrete variables are presented as proportions. Quantitative data were analysed by Student’s t-test, while categorical data were analysed by the chi squared test. The Kaplan–Meier method was used to evaluate differences in the TTP and OS rate between the two groups. Univariate analyses were implemented with the log-rank test. Variables with a *P* value of < 0.10 were entered into the multivariate analysis, which was performed using a Cox proportional hazard regression model.

## Results

### Study population and patient characteristics

From January 2010 to January 2020, a total of 436 patients received combination therapy as a treatment option, and 306 patients were excluded since they did not meet the study requirements, as highlighted in Fig. 1. Finally, a total of 130 patients were included in this study, of whom 96 were treated with early sequential RFA treatment after TACE, followed by palliative TACE treatment after subsequent progression. In addition, a total of 36 patients underwent 1–2 TACE procedures, then RFA treatment and finally palliative TACE treatment after tumour progression. The detailed clinical characteristics of the 130 patients are summarized in Table 1. The median follow-up period was 37 months (range 5.7–110.5 months) in the TACE + eRFA group and 33 months (range 14.4–103.9 months) in the TACE + lRFA group. In the TACE + lRFA group, 29 patients died during the observation period, whereas in the TACE + eRFA group, only 58 patients died.

**Table 1:** Baseline Characteristics

Characteristics	Early-RFA (N = 96) (No, %; Mean ± SD)	Late-RFA (N = 34) (No, %; Mean ± SD)	*P* value
**Gender**			0.67
Male	82 (85.4%)	28 (82.4%)	
Female	14 (14.6%)	6 (17.6%)	
**Age (years)**	55.53 ± 10.4	54.18 ± 11.1	0.52
**Bilirubin (µmol/L)**	20.3 ± 13.4	20.1 ± 11.8	0.94
**Albumin (g/L)**	37.8 ± 6.0	38.0 ± 5.4	0.87
**PT(s)**	14.3 ± 1.7	14.0 ± 1.6	0.31
**AST (µmol/L)**	48.0 ± 32.4	37.6 ± 29.9	0.11
**Maximal tumor diameter (cm)**			0.08
Mean ± SD	4.0 ± 2.8	5.0 ± 2.7	
Range	1.1–15.6	1.9–12.8	
1–3 cm	44 (45.8%)	12 (35.3%)	
3.1–5 cm	30 (31.3)	12 (35.3%)	
5.1–10 cm	17 (17.7%)	8 (23.5%)	
> 10 cm	5 (5.2%)	2 (5.9%)	
**No. of tumors**			0.20
Mean ± SD	1.18 ± 0.5	1.29 ± 0.5	
1	82 (85.4%)	24 (70.6%)	
2–3	14 (14.6%)	10 (29.4%)	
**Hepatitis**			0.56
Hepatitis B	83 (86.5%)	28 (82.4%)	
Other	13 (13.5%)	6 (17.6%)	
**α-Fetoprotein level**			0.95
> 400 ng/mL	26 (27.1%)	9 (26.5%)	
≤ 400 ng/ml	70 (72.9%)	25 (73.5%)	
**Child–Pugh score**			0.67
A	82 (85.4%)	28 (82.4%)	
B	14 (14.6%)	6 (17.6%)	
**BCLC**			0.17
A	44 (45.8%)	11 (32.4%)	
B	52 (54.2%)	23 (67.6%)	
**TACE sessions**	3.21 ± 2.6	5 ± 2.1	0.00

SD: Standard deviation; PT: Prothrombin time; AST: Aspartate aminotransferase; BCLC: Barcelona Clinic Liver Cancer; TACE: Transcatheter arterial chemoembolization

### Treatment response

The objective tumour regression rate was 89.6% in the TACE + eRFA group and 61.8% in the TACE + lRFA group (*P* = 0.000). In addition, the disease control rate was 94.8% in the TACE + eRFA group and 73.5% in the TACE + lRFA group (*P* = 0.002). Hence, compared with patients in the TACE + lRFA group, those in the TACE + eRFA group benefitted from an enhanced tumour response.

### Complications

Within the TACE + eRFA group, three patients had severe complications, for an incidence of 2.1%. Two patients developed a biloma following TACE, and one patient suffered from an intestinal perforation after RFA. There were two cases of severe complications in the TACE + lRFA group, for an incidence of 2.9%. A subcapsular haematoma in the liver was observed on CT in two patients. There was no significant difference in the incidence of major complications between the two groups (*P* = 0.84).

### OS

**Fig. 2:**
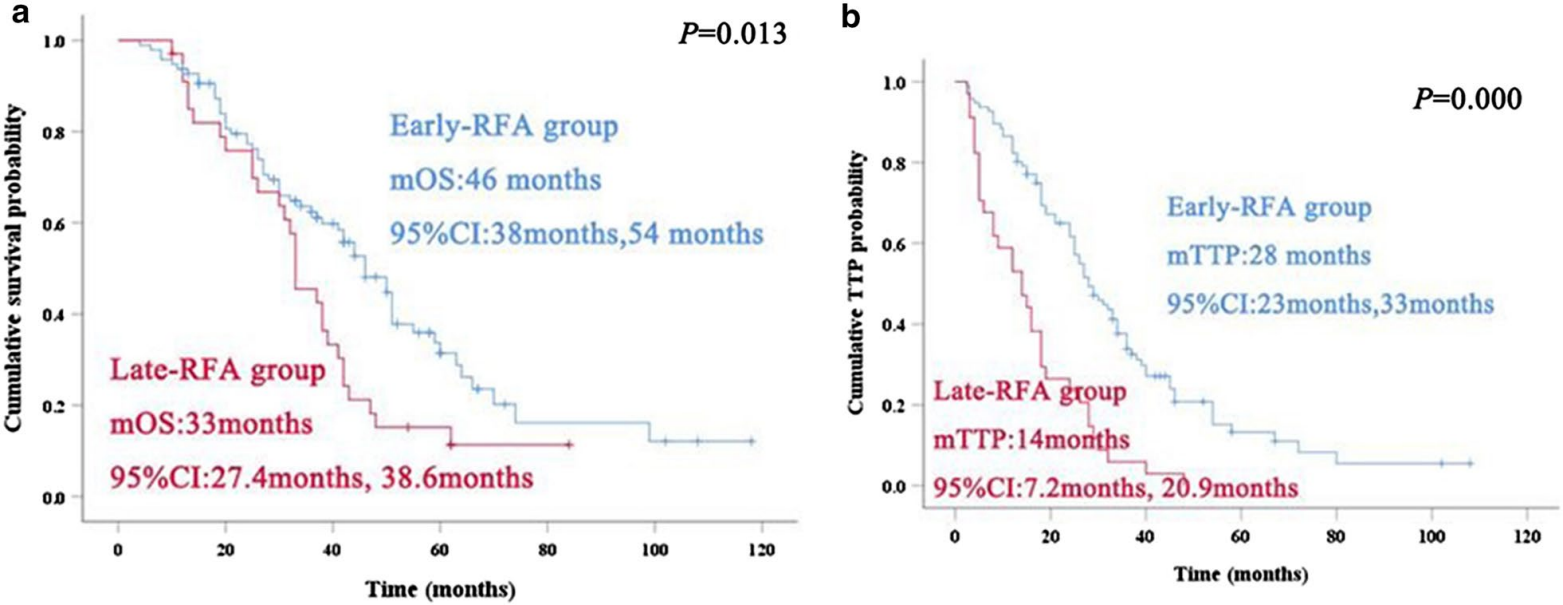
Kaplan–Meier curves for OS and TTP in the early-RFA (blue line) and late-RFA (red line) groups. **a** Cumulative survival probability (OS) was significantly longer (*p* = 0.013) in the early-RFA group (median OS: 46 months, 95% CI 38–54) than in the late-RFA group (median OS: 33 months, 95% CI 27.4–38.6). **b** The cumulative time to tumour progression (TTP) was significantly longer (*p* = 0.000) in the early RFA group (median TTP: 28 months, 95% CI 23–33 months) than in the late RFA group (median TTP: 14 months, 95% CI 7.2–20.9 months)

The median OS was 46 months (95%CI: 38.0–54.0) in the TACE + eRFA group and 33 months (95%CI: 27.4–38.6) in the TACE + lRFA group (*P* = 0.013) (Fig. 2a). Univariable analyses showed that the mean tumour size, alpha-fetoprotein (AFP) > 400 ng/mL, the Child–Pugh score, the BCLC stage and the therapy method were correlated with OS (Table 2). Consequently, through multivariable analysis (Table 3), we identified that the BCLC stage was an independent risk factor for OS (*P* = 0.038).

**Table 2:** Univariate analysis of prognostic factors for overall survival and time to progression

Variables	OS		TTP	
	HR (95% CI)	*P* value	HR (95% CI)	*P* value
**Gender**				
Male	1		1	
Female	1.213 (0.694, 2.119)	0.498	0.701 (0.415, 1.183)	0.183
**Age (years)**	1.003 (0.984, 1.023)	0.750	1.003 (0.986, 1.021)	0.715
**Bilirubin (µmol/L)**	1.003 (0.986, 1.020)	0.759	1.004 (0.990, 1.019)	0.548
**Albumin (g/L)**	1.021 (0.983, 1.060)	0.278	0.999 (0.967, 1.033)	0.972
**PT (s)**	0.943 (0.816, 1.089)	0.423	1.006 (0.891, 1.136)	0.921
**AST (µmol/L)**	1.004 (0.997, 1.010)	0.294	1.005 (0.999, 1.011)	0.116
**Tumor size**	1.179 (1.105, 1.257)	0.000	1.132 (1.066, 1.201)	0.000
**Tumor number**	1.326 (0.873, 2.015)	0.186	1.489 (1.032, 2.150)	0.033
**Hepatitis**				
Hepatitis B	1		1	
Other	0.861 (0.468, 1.584)	0.630	1.216 (0.704, 2.100)	0.483
**α-Fetoprotein level**				
≥ 400 ng/mL	1		1	
< 400 ng/ml	1.843 (1.169, 2.906)	0.008	1.738 (1.135, 2.662)	0.011
**Child–Pugh score**				
A	1		1	
B	0.575 (0.337, 0.980)	0.042	0.614 (0.373, 1.010)	0.055
**BCLC stage**				
A	1			
B	0.348 (0.217, 0.559)	0.000	0.472 (0.319, 0.700)	0.000
**TACE sessions**				
1	1		1	
2 or more	0.968 (0.901, 1.040)	0.372	1.050 (0.991, 1.114)	0.101
**Treatment method**				
Early-RFA	1			
Late-RFA	0.573 (0.365, 0.898)	0.015	0.351 (0.230, 0.535)	0.000

OS: Overall survival; TTP: Time to progression; HR: Hazard ratio; CI: Confidence interval; PT: Prothrombin time; AST: Aspartate aminotransferase; BCLC: Barcelona Clinic Liver Cancer; TACE: Transcatheter arterial chemoembolization; RFA: Radiofrequency ablation

**Table 3:** Multivariate analysis of prognostic factors for overall survival

Variables	HR (95% CI)	*P* value
**Tumor size**	1.090 (0.991, 1.199)	0.075
**α-Fetoprotein level**		
> 400 ng/mL	1	
≤ 400 ng/ml	1.446 (0.851, 2.456)	0.173
**Child–Pugh score**		
A	1	
B	0.587 (0.340, 1.013)	0.056
**BCLC stage**		
A		
B	0.544 (0.306, 0.968)	0.038
**Treatment method**		
Early-RFA	1	
Late-RFA	0.659 (0.415, 1.049)	0.079

HR: Hazard ratio; CI: Confidence interval; BCLC: Barcelona Clinic Liver Cancer; RFA: Radiofrequency ablation

### TTP

The median TTP was 28 months (95%CI 28.0–33.0) in the TACE + eRFA group and 14 months (95% CI 7.2.0–20.9) in the TACE + lRFA group (*P* < 0.001) (Fig. 2b). Univariable analyses indicated that the mean tumour size, the tumour number, AFP > 400 ng/mL, the BCLC stage and TACE + eRFA were correlated with the TTP (Table 2). Multivariable analysis revealed that TACE + eRFA was associated with an enhanced TTP (Table 4).

**Table 4:** Multivariate analysis of prognostic factors for time to progression

Variables	HR (95% CI)	*P* value
**Tumor size**	1.036 (0.939, 1.143)	0.480
**Tumor number**	1.464 (0.962, 2.230)	0.075
**α-Fetoprotein level**		
> 400 ng/mL	1	
≤ 400 ng/ml	1.580 (0.949, 2.631)	0.079
**Child–Pugh score**		
A	1	
B	0.623 (0.370, 1.048)	0.074
**BCLC stage**		
A		
B	0.662 (0.398, 1.102)	0.113
**Treatment method**		
Early-RFA	1	
Late-RFA	0.397 (0.252, 0.625)	0.000

HR: Hazard ratio; CI: Confidence interval; BCLC: Barcelona Clinic Liver Cancer; RFA: Radiofrequency ablation

## Discussion

The combination of TACE and RFA has several theoretical advantages over RFA or TACE alone [25, 26]. First, the TACE procedure can reduce the heat-sink effect by inhibiting blood flow in the hepatic artery, thereby expanding the ablation area [27]. In addition, the inclusion of TACE renders the evaluation of ablative margins less challenging and enhances the control of satellite lesions [28]. Conversely, RFA as a radical treatment can lead to a better tumour response, and an enlarged ablation boundary can reduce the formation of a collateral tumour circulation [29–31]. Following repeated TACE procedures, TACE failure/refractory effects often occur [19], leading to a poor tumour response and damaged liver function, eventually causing the upregulation of vascular endothelial growth factor (VEGF) and hypoxia inducible factor (HIF) expression and ultimately reducing the OS rate. Early sequential RFA treatment can cause complete tumour necrosis and inhibit the upregulation of VEGF and HIF expression after hypoxia due to TACE, which reduces local progression and improves patient survival [32].

Presently, there is a lack of relevant literature on the timing of combination therapy. Most studies recommend RFA treatment within two weeks post-TACE [23–27] to better restore liver function. According to one school of thought, a time interval of 0–2 days is sufficient to allow for liver functional reserve recovery in patients with cirrhosis [25]. However, others believe that a shorter time interval deteriorates patient liver function. A period of 3–5 weeks is the optimal time interval for suitable patients to undergo combination therapy, with a well-balanced clinical prognosis [33].

**Fig. 3:**
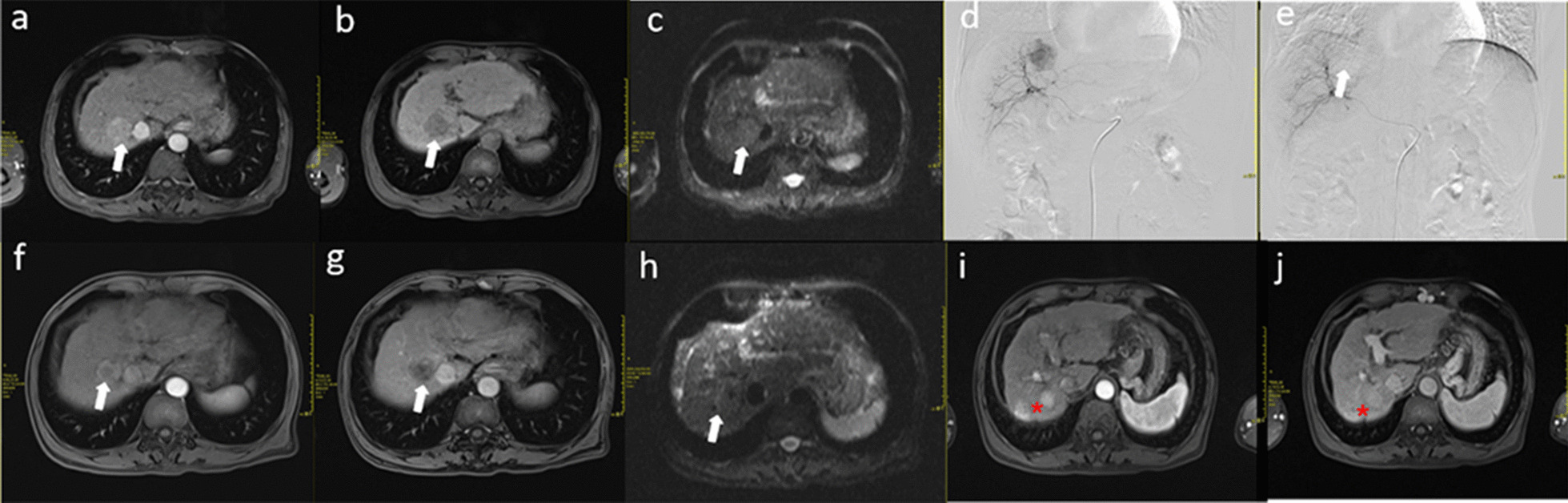
MRI and digital subtraction angiography (DSA) images of a patient receiving early RFA (5 days) after TACE treatment. **a**–**c** The lesion (white arrow) showed a rapid increase in signal intensity during the hepatic arterial phase and the portal venous phase; the entire lesion (white arrow) showed rapid washout of the contrast material and became hypointense compared with the surrounding liver parenchyma. Diffusion-weighted imaging (DWI) showed the dispersion constant. **d**, **e** DSA images before and after the initial TACE treatment; the tumour staining disappeared (white arrow). **f**–**h** One month after combined treatment, the entire lesion (white arrow) showed necrotic foci in the centre, and DWI showed no dispersion constant. **i**–**j** After 48 months, there were two lesions in the liver, indicating long-term recurrence (red star sign)

**Fig. 4:**
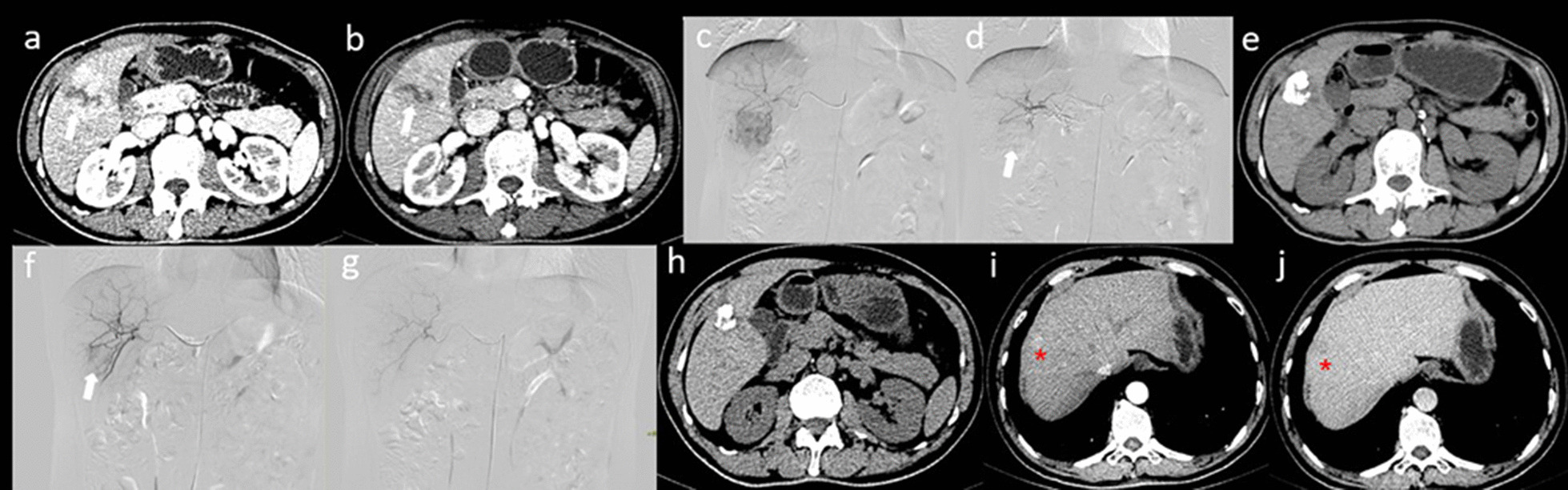
CT and DSA images of a patient receiving 1 TACE treatment before combination therapy. **a**, **b** Axial CT showed a typical HCC enhancement pattern, including “wash in” in the arterial phase, “wash out” in the portal phase (white arrow) in the marginal zone, and necrotic foci in the centre. **c**, **d** DSA images before and after the initial TACE treatment; the tumour staining disappeared (white arrow). **e** Re-examination after one and a half months showed that the tumour had decreased in size and that viable tumours were present on the edges. **f**, **g** During the second TACE treatment, the tumour still showed staining (white arrow), indicating that the tumour blood vessels were recanalized and that the tumour was alive. The staining disappeared after treatment. **h** Re-examination after the combination therapy showed that the tumour had decreased in size and that there were no live foci. **i**, **j** Twelve months after late combination therapy, there was recurrence in the liver (red star sign)

In this study, due to various reasons, some patients failed to undergo combination therapy within two weeks; they underwent 1–2 TACE procedures before treatment and continued to receive palliative TACE treatments after progression. Our results demonstrate that early sequential RFA treatment (Fig. 3) provides better local control, reduced the number of TACE procedures, and prolongs both TTP and survival compared with late combined treatment (Fig. 4). Consequently, if RFA is available, it should actively be recommended to HCC patients as soon as possible. This study indicates that early combined therapy can improve the therapeutic efficacy in patients with HCC.

Similar to other studies [12, 32, 34], our study revealed that the tumour size, AFP level, Child–Pugh score, and BCLC stage are prognostic factors for OS and TTP. Notably, early sequential RFA treatment was an independent prognostic factor for TTP and is generally safe; there was no significant difference in the incidence of major complications between the two groups.

However, the sample size was small, and the retrospective, non-randomized experimental design was the main flaw of the study. Consequently, a prospective, randomized controlled trial is necessary to verify our results. In addition, due to the limited number of samples, no stratified analysis was conducted.

## Conclusions

In essence, early sequential RFA treatment in patients with early- and mid-stage HCC can improve local tumour control and improve clinical outcomes, such as OS and TTP, while reducing the frequency of TACE treatment. In clinical practice, for HCC patients initially treated with TACE, it is recommended to combine RFA as soon as possible to improve the long-term survival of individual HCC patients.

## Data Availability

The datasets used and/or analyzed during the current study are available from the corresponding author on reasonable request.

## Abbreviations

BCLC: Barcelona Clinic Liver Cancer
RFA: Radiofrequency ablation
eRFA: Early radiofrequency ablation
lRFA: Late radiofrequency ablation
mRECIST: The modified Response Evaluation Criteria in Solid Tumors
CR: Complete response
PR: Partial response
SD: Stable disease
PD: Progressive disease
VEGF: Vascular endothelial growth factor
HIF: Hypoxia inducible factor
AFP: Alpha-fetoprotein
CT: Computed tomography
EASL: European Association for the Study of the Liver
AASLD: American Association for the Study of Liver Disease: Hepatocellular carcinoma
MR: Magnetic resonance
OS: Overall survival
TTP: Time to tumour progression
TACE: Transarterial chemoembolization
